# Bone Fracture Risk is Not Associated with the Use of Glucagon-Like Peptide-1 Receptor Agonists: A Population-Based Cohort Analysis

**DOI:** 10.1007/s00223-015-9993-5

**Published:** 2015-04-17

**Authors:** Johanna H. M. Driessen, Ronald M. A. Henry, Hein A. W. van Onzenoort, Arief Lalmohamed, Andrea M. Burden, Daniel Prieto-Alhambra, Cees Neef, Hubert G. M. Leufkens, Frank de Vries

**Affiliations:** Division of Pharmacoepidemiology and Clinical Pharmacology, Utrecht Institute of Pharmaceutical Sciences, Utrecht University, Utrecht, The Netherlands; Care and Public Health Research Institute (CAPHRI), Maastricht University, Maastricht, The Netherlands; Department of Clinical Pharmacy and Toxicology, Maastricht University Medical Centre+, Maastricht, The Netherlands; Department of Medicine, Maastricht University Medical Centre+, Maastricht, The Netherlands; Cardiovascular Research Institute Maastricht, Maastricht University Medical Centre+, Maastricht, The Netherlands; Department of Pharmacy, Radboud University Nijmegen Medical Centre, Nijmegen, The Netherlands; Department of Clinical Pharmacy, University Medical Center Utrecht, Utrecht, The Netherlands; Nuffield Department of Orthopaedics, Rheumatology and Musculoskeletal Sciences, Oxford NIHR Musculoskeletal Biomedical Research Unit, University of Oxford, Oxford, UK; MRC Epidemiology Lifecourse Unit, Southampton General Hospital, Southampton, UK

**Keywords:** GLP1-ra, Fracture, Diabetes mellitus type 2, Cohort study, CPRD

## Abstract

Glucagon-like Peptide-1 receptor agonists (GLP1-ra) are a relatively new class of anti-hyperglycemic drugs which may positively affect bone metabolism and thereby decrease (osteoporotic) bone fracture risk. Data on the effect of GLP1-ra on fracture risk are scarce and limited to clinical trial data only. The aim of this study was to investigate, in a population-based cohort, the association between the use of GLP1-ra and bone fracture risk. We conducted a population-based cohort study, with the use of data from the Clinical Practice Research Datalink (CPRD) database (2007–2012). The study population (*N* = 216,816) consisted of all individuals with type 2 diabetes patients with at least one prescription for a non-insulin anti-diabetic drug and were over 18 years of age. Cox proportional hazards models were used to estimate the hazard ratio of fracture in GLP1-ra users versus never-GLP1-ra users. Time-dependent adjustments were made for age, sex, lifestyle, comorbidity and the use of other drugs. There was no decreased risk of fracture with current use of GLP1-ra compared to never-GLP1-ra use (adjusted HR 0.99, 95 % CI 0.82–1.19). Osteoporotic fracture risk was also not decreased by current GLP1-ra use (adjusted HR 0.97; 95 % CI 0.72–1.32). In addition, stratification according to cumulative dose did not show a decreased bone fracture risk with increasing cumulative GLP1-ra dose. We showed in a population-based cohort study that GLP1-ra use is not associated with a decreased bone fracture risk compared to users of other anti-hyperglycemic drugs. Future research is needed to elucidate the potential working mechanisms of GLP1-ra on bone.

## Introduction

In individuals with type 2 diabetes, the risk of bone fracture is increased compared to individuals without type 2 diabetes [1]. This increased risk might be associated with the pathobiology of type 2 diabetes itself, although the underlying mechanisms remain largely unknown [2]. Alternatively, it has been suggested that this increased bone fracture risk is a consequence of the kind of therapeutic regimen initiated to combat hyperglycemia [3]. For instance, thiazolidinediones [4–6] have shown to increase fracture risk. It has been suggested that human recombinant insulins [7] also increase bone fracture risk, whereas metformin might actually decrease it [8].

Glucagon-like peptide-1 receptor agonists (GLP1-ra) are a relative new class of anti-hyperglycemic drugs, which may positively affect bone metabolism [9–11] and decrease bone fracture risk. However, a recent meta-analysis, based upon randomized clinical trial data, in which bone fractures were not a primary outcome, did not show a decreased bone fracture risk [12]. Thus, the aim of the present study was to investigate, in a large population-based cohort study, the association between GLP1-ra use and the risk of bone fractures in individuals with type 2 diabetes.

## Methods

### Data Source

Data were obtained from the British Clinical Practice Research Datalink [CPRD; previously the General Practice Research Database (GPRD); see (www.CPRD.com)]. The CPRD contains computerized medical records of 625 primary care practices in the United Kingdom (UK), and patients represent 8 % of the UK population. Data have been collected since 1987 and include, amongst others, demographic information, prescription details, data on morbidity and mortality, preventive care provided and specialist referrals. Data in the CPRD have been shown to be accurate and valid [13]. Particularly, with regard to the main outcome of the present study, fractures have been validated in over 90 % of all cases [14].

### Study Population

We conducted a population-based cohort study. The population consisted of all patients with at least one prescription for a non-insulin anti-hyperglycemic drug (NIAD) and who were aged 18+ during the period of valid CPRD data collection [15]. Cohort entry was defined as the date of first prescription for GLP1-ra, identified between June 13th, 2007 and August 31, 2012. The index date was defined as the date of the first NIAD prescription; thereby the study population was a mix of incident and prevalent NIAD users. Patients were followed from the index date to the end of data collection, date of transfer of the patient out of the practice area, patient’s death, or fracture types of interest, whichever came first.

### Exposure

Follow-up time was divided into intervals based on NIAD and insulin prescriptions. Thus a new interval was created for every prescription. When there was a washout period of 90 days, an interval was classified as “past use”, until end of follow-up, or a new anti-hyperglycemic drug prescription, whichever came first. In all other circumstances, an interval was classified as “current use”. If no GLP1-ra was prescribed during follow-up, person-time was classified as never use of GLP1-ra.

All GLP1-ra exposed intervals were classified, according to the time since the most recent prescription, as current (1–90 days), recent (91–180 days), or past (over 180 days) GLP1-ra use. At every current use interval, the cumulative prescribed GLP1-ra dosage, in exenatide dose equivalents, was reviewed and divided by the GLP1-ra treatment time (difference in time between the start of the first and last GLP1-ra prescription) to estimate the average daily GLP1-ra dose. Defined daily doses were used to calculate the exenatide dose equivalents [16].

### Outcomes

Patients were followed from the index date to the end of data collection, date of transfer of the patient out of the practice area, patient’s death, or fracture types of interest, whichever came first. Fractures were classified with the use of READ codes [17] into hip, radius and (or) ulna, vertebral, humerus and other fractures. A major osteoporotic fracture was defined according to the WHO definition as a fracture of the hip, humerus, vertebral or radius/ulna [18].

### Other Variables

The presence of risk factors for bone fractures was assessed by reviewing the computerized medical records for any record of any risk factors for bone fractures prior to the start of an interval. The following potential confounders were determined at baseline: sex, body mass index (BMI), smoking status and alcohol use. All other potential confounders that were considered in this study were determined time-dependent (i.e. at the start of each interval): age, falls in 7–12 months before the start of an interval, a history of chronic obstructive pulmonary disease (COPD), a previous fracture, rheumatoid arthritis, hypothyroidism, hyperthyroidism, cancer, retinopathy, neuropathy, secondary osteoporosis (hypogonadism or early menopause) and congestive heart failure. The most recent HbA1c value up to 1 year prior to the start of an interval was determined. The following drug prescriptions, in the 6 months prior to the start of an interval, were considered potential confounders; oral glucocorticoids [19], cholesterol lowering drugs, antidepressants [20], anxiolytics or hypnotics [21], antipsychotics, anti-Parkinson drugs [22], antihypertensives (beta-blockers, thiazide diuretics, renin-angiotensin-aldosterone-system (RAAS) inhibitors, calcium channel blockers and loop diuretics), antiarrhythmics, hormone replacement therapy, calcium, bisphosphonates, vitamin D, raloxifene, strontium ranelate, calcitonin and parathyroid hormone.

### Statistical Analyses

Regression analysis with Cox proportional hazards models (SAS 9.2, PHREG procedure) was used to estimate bone fracture risk of GLP1-ra users (current, recent or past) compared to never-GLP1-ra users. Current GLP1-ra use was further stratified to age and gender, and the main analyses were repeated for major osteoporotic bone fracture risk. In a series of further analyses, current GLP1-ra use was stratified according to type of GLP1-ra (i.e. exenatide and liraglutide), daily and cumulative dose. As a sensitivity analysis, the person-time for thiazolidinediones (TZD) was excluded from the reference group and analysed as a separate group.

In all analyses, potential confounders were included if they independently changed the beta-coefficient for current GLP1-ra exposure by at least 5 %, or when consensus about inclusion existed within the team of researchers, supported by clinical evidence from the literature.

## Results

### Study Population and Follow-up

In total, 216,816 individuals were included in the present study, of which 8,354 used GLP1-ra (either current, recent or past). The characteristics of the study population are presented in Table 1. On average, GLP1-ra users were younger than never-GLP1-ra users (53.5 vs. 61.0 years), and had a higher BMI (37.5 vs. 31.0). The median duration of follow-up time (from start of follow-up to end of data collection) was 5.1 years [Interquartile Range (IQR): 3.6–5.2 years] for GLP1-ra users and 3.6 years [IQR: 1.6–5.2 years] for never-GLP1-ra users.

**Table 1:** Baseline characteristics of GLP1-ra users and never-GLP1-ra users

Characteristics	GLP1-ra users^a^ *N* = 8354	Never-GLP1-ra users^b^ *N* = 208,462
Median [IQR] follow-up time (years)	5.1 [3.6–5.2]	3.6 [1.6–5.2]
Median [IQR] duration of actual GLP1-ra use (years)	1.7 [0.8–2.7]	n/a
Women	3904 (46.7)	98,614 (47.3)
Age		
Mean age at index date, (years) (SD)	53.5 (10.5)	61.0 (15.1)
18–49	2952 (35.3)	43,574 (20.9)
50–59	2909 (34.8)	41,717 (20.0)
60–69	1986 (23.8)	54,865 (26.3)
70–79	481 (5.8)	46,907 (22.5)
80+	26 (0.3)	21,399 (10.3)
BMI		
Mean BMI at index date, (SD)	37.5 (7.1)	31.0 (6.5)
<20.0 kg/m^2^	3 (0.0)	2759 (1.3)
20.0–24.9 kg/m^2^	84 (1.0)	26,969 (12.9)
25.0–29.9 kg/m^2^	936 (11.2)	67,550 (32.4)
30.0–34.9 kg/m^2^	2369 (28.4)	57,826 (27.7)
≥35.0 kg/m^2^	4921 (58.9)	48,016 (23.0)
Missing	41 (0.5)	5342 (2.6)
HbA1c (%)		
Mean (SD)	8.6 (1.9)	8.0 (1.8)
Missing	2915 (34.9)	88,937 (42.7)
Smoking status		
Never	3949 (47.3)	103,970 (49.9)
Current	1945 (23.3)	41,110 (19.7)
Ex	2458 (29.4)	62,287 (29.9)
Missing	2 (0.0)	1095 (0.5)
Alcohol use		
No	2417 (28.9)	60,278 (28.9)
Yes	5638 (67.5)	136,373 (65.4)
Missing	299 (3.6)	11,811 (5.7)
Falls (7–12 months before)	52 (0.6)	2141 (1.0)
History of diseases		
Fracture	52 (0.6)	1342 (0.6)
Hyperthyroidism	64 (0.8)	2014 (1.0)
Hypothyroidism	719 (8.6)	16,413 (7.9)
COPD	345 (4.1)	11,585 (5.6)
Congestive heart failure	244 (2.9)	8846 (4.2)
Cancer	1626 (19.5)	45,626 (21.9)
Rheumatoid arthritis	109 (1.3)	3633 (1.7)
Retinopathy	1284 (15.4)	24,954 (12.0)
Secondary osteoporosis	895 (10.7)	18,571 (8.9)
Neuropathy	768 (9.2)	15,652 (7.5)
Drug use within 6 months before		
Metformin	7367 (88.2)	171,699 (82.4)
Sulfonylurea derivatives	4589 (54.9)	54,874 (26.3)
Thiazolidinediones	2108 (25.2)	20,293 (9.7)
Insulin	2238 (26.8)	22,556 (10.8)
DPP4 inhibitors	144 (1.7)	1374 (0.7)
Glucocorticoids	439 (5.3)	12,077 (5.8)
Statins	5803 (69.5)	113,343 (54.4)
Antiarrhythmics	125 (1.5)	3336 (1.6)
Antidepressants	2075 (24.8)	32,995 (15.8)
Anti-Parkinson drugs	24 (0.3)	1040 (0.5)
Antipsychotics	157 (1.9)	4343 (2.1)
Anxiolytics	658 (7.9)	14,752 (7.1)
Hypnotics	437 (5.2)	10,242 (4.9)
Antihypertensives	5651 (67.6)	120,188 (57.7)
Bisphosphonates	87 (1.0)	5569 (2.7)
Raloxifene	5 (0.1)	298 (0.1)
Vitamin D	42 (0.5)	1161 (0.6)
Calcium	145 (1.7)	8263 (3.9)
Strontium	0 (−)	124 (0.1)
PTH/calcitonin	0 (−)	0 (−)
Hormone replacement therapy	71 (0.8)	835 (0.4)

Data are number (%) of patients, unless stated otherwise

*SD* indicates standard deviation, *BMI* body mass index, *COPD* chronic obstructive pulmonary disease, *PTH* parathyroid hormone, *GLP1-ra* glucagon-like peptide-1 receptor agonist, *IQR* interquartile range, *DPP4* dipeptidyl peptidase 4

^a^GLP1-ra users are patients who had at least 1 GLP1-ra prescription during follow-up

^b^Never-GLP1-ra users are patients who had at least 1 NIAD prescription other than GLP1-ra, during follow-up

### Current, Recent or Past GLP1-ra Use and the Risk of Bone Fracture

After adjusting for confounders, the risk for bone fractures with current GLP1-ra, compared to never-GLP1-ra users, was [Hazard Ratio (HR) and (95 %CI)] 0.99 (0.82–1.19), with recent GLP1-ra use: 1.19 (0.66–2.14) and with past GLP1-ra use: 1.38 (1.03–1.84), Table 2. Stratification of current GLP1-ra by GLP1-ra type resulted in an adjusted (adj.) HR of 0.90 (0.69–1.17) with use of liraglutide and an adj. HR of 1.08 (0.84–1.38) with exenatide use.

**Table 2:** Risk of bone fracture in GLP1-ra users compared with never-GLP1-ra users

	No. of fractures *N* = 9340^a^	Fracture IR (/1000 PY)	Age/sex adjusted HR (95 % CI)	Adjusted HR^c^ (95 % CI)
GLP1-ra exposure				
Never use^b^	8449	12.9	Reference	Reference
Past use	47	16.1	1.48 (1.11–1.95)^*^	1.38 (1.03–1.84)^*^
Recent use	11	13.4	1.22 (0.69–2.14)	1.19 (0.66–2.14)
Current use	122	10.6	1.00 (0.83–1.20)	0.99 (0.82–1.19)
By GLP1-ra type				
Liraglutide	57	9.5	0.91 (0.70–1.18)	0.90 (0.69–1.17)
Exenatide	65	11.8	1.09 (0.85–1.40)	1.08 (0.84–1.38)
By sex^d^				
Men	53	8.5	1.02 (0.78–1.35)	1.01 (0.76–1.33)
Women	69	13.1	0.98 (0.77–1.25)	0.96 (0.75–1.22)
By age^e^				
18–49	22	7.6	0.88 (0.57–1.35)	0.80 (0.52–1.25)
50–59	33	8.5	0.83 (0.58–1.19)	0.80 (0.56–1.15)
60–69	54	15.0	1.48 (1.12–1.95)	1.38 (1.04–1.83)
70+	13	11.3	0.60 (0.35–1.04)	0.68 (0.39–1.18)

Current GLP1-ra use: most recent prescription within 90 before start of an interval

Recent GLP1-ra use: most recent prescription within 91–180 before start of an interval

Past GLP1-ra use: most recent prescription over 180 days before start of an interval

All models are corrected for DPP4-I use

*HR* indicates hazards ratio, *CI* confidence interval, IR, incidence rate, *GLP1-ra* glucagon-like peptide-1 receptor agonist, *DPP4-I* dipeptidyl peptidase-4 inhibitor, *BMI* body mass index

* Statistically significant, (*P* < 0.05)

^a^Past NIAD use not shown, therefore, the total number of fractures does not add up

^b^Never GLP1-ra use does not include use of DPP4-I

^c^Adjusted for sex, age, BMI, smoking status, HbA1c, retinopathy, neuropathy, secondary osteoporosis, and the use of glucocorticoids, cholesterol lowering drugs, hypnotic/anxiolytic drugs and antidepressants

^d^Models not statistically adjusted for sex

^e^Models not statistically adjusted for age

When stratified by sex, bone fracture risk for current male GLP1-ra users was 1.01 (0.76–1.33) and for current female GLP1-ra users was 0.96 (0.75–1.22), Table 2. After stratification by age group (i.e. 18–49, 50–59, 60–69 and ≥70 years), the risk for bone fractures among patients with current GLP1-ra use aged 18–49 was 0.80 (0.52–1.25), aged 50–59 was 0.80 (0.56–1.15), aged 60–69 was 1.38 (1.04–1.83) and aged 70 years or older was 0.68 (0.39–1.18), Table 2.

As compared to never-GLP1-ra use, the risk for major osteoporotic fractures with current GLP1-ra use was 0.97 (0.72–1.32); with recent GLP1-ra use, 1.13 (0.42–3.02) and with past GLP1-ra use, 1.04 (0.61–1.76), Table 3, (detailed data not shown). Current GLP1-ra use was not associated with a decreased bone fracture risk for other fracture types, Table 3.

**Table 3:** Risk of bone fracture in current GLP1-ra users stratified to fracture type

Fracture sites	No. of fractures	Fracture IR (/1000 PY)	Age/sex adjusted HR (95 % CI)	Adjusted HR (95 % CI)
Major osteoporotic fracture				
Never-GLP1-ra use^a^	4373	6.1	Reference	Reference
Current GLP1-ra use	44	3.7	0.97 (0.71–1.31)	0.97 (0.72–1.32)^b^
Hip fracture				
Never-GLP1-ra use^a^	1383	1.9	Reference	Reference
Current GLP1-ra use	2	0.2	0.26 (0.06–1.03)	0.27 (0.07–1.08)^c^
Radius/ulna fracture				
Never-GLP1-ra use^a^	1442	2.0	Reference	Reference
Current GLP1-ra use	15	1.3	1.78 (0.47–1.32)	0.82 (0.48–1.37)^d^
Vertebral fracture				
Never-GLP1-ra use^a^	513	0.7	Reference	Reference
Current GLP1-ra use	8	0.7	1.59 (0.78–3.24)	1.64 (0.80–3.37)^e^

Current GLP1-ra use: most recent prescription within 90 before start of an interval

All models are corrected for DPP4-I use

*HR* indicates hazards ratio, *CI* confidence interval, *IR* incidence rate, *GLP1-ra* glucagon-like peptide-1 receptor agonist, *DPP4-I* dipeptidyl peptidase-4 inhibitor, *BMI* body mass index

* Statistically significant, (*P* < 0.05)

^a^Never-GLP1-ra use does not include use of DPP4-I

^b^Adjusted for (f), and history of congestive heart failure, use of cholesterol lowering drugs, antidepressants, hypnotics/anxiolytics, calcium and anti-osteoporotic drugs^g^

^c^Adjusted for (f), and the use of glucocorticoids, antidepressants, and falls 7–12 months before index date

^d^Adjusted for (f), and the use of cholesterol lowering drugs, antidepressants, hypnotics/anxiolytics, anti-osteoporotic drugs^g^ and glucocorticoids

^e^Adjusted for (f), and history of congestive heart failure, use of cholesterol lowering drugs, antidepressants, hypnotics/anxiolytics and glucocorticoids

^f^Sex, age, BMI, smoking status, HbA1c, retinopathy, neuropathy and secondary osteoporosis

^g^Use of bisphosphonates, raloxifene, strontium ranelate or PTH/calcitonin

### Bone Fracture Risk and Current GLP1-ra Use Stratified According to Dosage

If current GLP1-ra use was stratified according to cumulative dose (i.e. 0–2.7 mg, 2.8–5.4 mg, 5.5–8.2 mg and ≥8.3 mg exenatide dose equivalents) the results showed that, as compared to never-GLP1-ra users, bone fracture risk for 0–2.7 mg was 1.02 (95 % CI 0.75–1.40), for 2.7–5.5 mg, 0.89 (0.60–1.34); for 5.5–8.2 mg, 0.94 (0.58–1.52) and for ≥8.3 mg, 1.03 (0.75–1.41). When stratified according to current GLP1-ra average daily dose use (i.e. missing, 0–15 mcg, 16–20 mcg and >20 mcg exenatide dose equivalents), the results were not substantially altered, Table 4.

**Table 4:** Risk of bone fracture in current GLP1-ra users compared to never-GLP1-ra users, stratified by average and cumulative DDD exposure

	No. of fractures	Fracture IR (/1000 PY)	Age/sex adjusted HR (95 % CI)	Adjusted HR (95 % CI)^b^
Never-GLP1-ra use^a^	8449	12.9	Reference	Reference
Current GLP1-ra use				
By average DDD exposure (in exenatide equivalents)				
0–15 mcg	48	16.9	1.05 (0.79–1.40)	1.03 (0.78–1.38)
16–20 mcg	37	8.7	0.82 (0.59–1.14)	0.82 (0.59–1.13)
≥21 mcg	28	14.3	1.29 (0.89–1.88)	1.27 (0.88–1.85)
No dose	9	9.8	0.91 (0.48–1.76)	0.90 (0.47–1.73)
By cumulative DDD exposure (in exenatide equivalents)				
0–2.7 mg	41	11.3	1.03 (0.76–1.41)	1.02 (0.75–1.40)
2.8–5.4 mg	24	9.4	0.90 (0.60–1.35)	0.89 (0.60–1.34)
5.5–8.2 mg	17	9.9	0.95 (0.59–1.53)	0.94 (0.58–1.52)
≥8.3 mg	40	11.0	1.05 (0.77–1.44)	1.03 (0.75–1.41)

All models are corrected for DPP4-I use

*HR* hazards ratio, *CI* confidence interval, *IR* incidence rate, *GLP1-ra* glucagon-like peptide-1 receptor agonist, *DPP4-I* dipeptidyl peptidase-4 inhibitor

^a^NIAD past use, GLP1-ra recent and GLP1-ra past use not shown. Never-GLP1-ra use does not include use of DPP4-I

^b^Adjusted for sex, age, bmi, smoking status, HbA1c, history of secondary osteoporosis, retinopathy and neuropathy, use of glucocorticoids, cholesterol lowering drugs, hypnotic/anxiolytic drugs and antidepressants

### Sensitivity Analyses

As TZD use has been associated with an increased risk of fracture, we additionally excluded for the main analysis all TZD exposed person-time from the reference group and analysed it as a separate group. This did not substantially change the results of GLP1-ra use (adj. HR with current GLP1-ra use; 1.03 (0.86–1.24), with recent GLP1-ra use; 1.19 (0.66–2.15) and with past GLP1-ra use; 1.38 (1.03–1.84)).

## Discussion

The results of the present population-based study show that (osteoporotic) bone fracture risk was not decreased by GLP1-ra use. In addition, stratification according to cumulative dose did not show a decreased risk of bone fracture with increasing cumulative GLP-1 dose. The results of the present study thereby do not support the hypothesis that GLP1-ra use may reduce bone fracture risk in individuals with type 2 diabetes. The results of our study add to the field population-based data and are indirectly supported by a clinical trial on the effect of exenatide on markers of bone remodelling and calcium homeostasis, which failed to show a positive effect [23]. Our study is thereby in line with a recent meta-analysis [12] done on randomized clinical trial data. Another more recent meta-analysis also showed no association between use of GLP1-ra and fracture risk [24]. However, after stratification to GLP1-ra type, they found a decreased risk of fracture with use of liraglutide and an increased risk of fracture with use of exenatide. The results of our study did not show a decreased or increased risk after stratification by GLP1-ra type. It has to be taken into account, however, that the included studies of these meta-analyses were not designed to investigate fracture risk and that fractures were not routinely registered. The results of our study are also in keeping with the results of a large cohort study on the use of dipeptidyl peptidase-4 inhibitors (DPP4-I) and fracture risk which also did not show a decreased fracture risk [25].

The pathways through which GLP1-ra’s may act on bone metabolism are not fully elucidated, but it has been suggested that GLP1-ra’s may, either directly or indirectly, shift the balance in bone homeostasis towards bone formation [26], via receptor coupling on osteoblasts [27] and (or) thyroid C cells [28, 29]. Alternatively, it has been suggested that GLP1-ra may increase calcitonin concentration [29, 30] and decrease sclerostin which both may inhibit bone formation [31]. Nevertheless, it remains to be determined whether such mechanisms may also be operative in humans. Interestingly, a recent meta-analysis of clinical trial data on the use of DPP4-I did show a 40 % reduction in the risk of bone fracture [32]. The latter brings forward the hypothesis that any effects on bone metabolism by DPP4-I might be independent from the direct effect of GLP1 on bone, despite the pharmacodynamics through which they are linked [3]. However, the underlying mechanisms between anti-hyperglycemic drug use along the GLP-1/DPP4-I axis and bone fracture risk in type 2 diabetes in humans remain complex.

Unexpectedly, our results showed a 1.4-fold increase in bone fracture risk for past GLP1-ra users (GLP1-ra use had discontinued ≥180 days) and for patients aged 60–69 compared to never-GLP1-ra users. As any plausible underlying mechanism seems missing, we consider these results as a play of chance.

Our study had several strengths. Firstly, the results were based upon population-based data that may have prevented, at least partially, selection bias as compared to randomized clinical trials in which only patients meeting specific inclusion criteria are able to participate. Secondly, bone fractures are often not primary end-points in clinical trials, thus their registration may be inadequate. The current study, however, was able to partially circumvent this potential bias, as over 90 % of all fractures have been clinically validated in the CPRD [14]. Finally, all participants were extensively clinically characterized which allowed us to take a series of potential confounders, including prior medical history, into account. In addition, we were able to adjust for neuropathy, retinopathy and HbA1c with which we tried to capture disease severity. In particular, HbA1c could act as a potential confounder of the association between GLP1-ra use and bone fracture risk in individuals with type 2 diabetes. However, it is acknowledged that some residual confounding may still be present.

When interpreting the results, a couple of limitations are worth mentioning. First, an important consideration, in light of the discussion on GLP1-ra use and bone fracture risk, in terms of time effect, is the average duration of GLP1-ra use. In our analysis, the median duration of actual GLP1-ra use was 1.2 years (from first GLP1-ra prescription until last GLP1-ra prescription), and this might be relatively short. For bisphosphonate use, it has been shown that bone fracture risk starts to decrease after 1–1.5 years of use [33, 34]. Even for the highest cumulative dose group (i.e. ≥8.3 mg), which could be equivalent to 1 DDD GLP1-ra per day during at least 1.5 years, we did not show a decrease in bone fracture risk. However, the time-window for GLP1-ra to exert an effect on bone fracture risk is not yet determined. Second, the risk of fracture is known to increase with age [35], yet we identified that patients who used GLP1-ra were slightly younger at baseline, compared to never-GLP1-ra users. Thus, this might have masked the protective effect of GLP1-ra on risk of bone fracture. Yet, our age-stratified analyses did not show a protective effect of GLP1-ra use on bone fracture risk. Third, GLP1-ra's are selectively prescribed to patients with an high BMI, which might have influenced the results. High BMI has been associated with a lower risk of fracture [36], and this might even strengthen the protective effect of GLP1-ra. However, we could not show a protective effect of GLP1-ra on fracture risk. Fourth, after stratification of the analyses to specific fracture types, the number of fractures within the current GLP1-ra group became low, and therefore, the results should be interpreted with caution.

In summary, we showed in a population-based cohort study that GLP1-ra use is not associated with a decreased risk of bone fracture as compared to users of other anti-hyperglycemic drugs. Future research is needed to elucidate the working mechanisms of the complex GLP-1/DPP-4 axis and to investigate the time-window of GLP1-ra to exert an effect on bone fracture risk.
